# ArkMAP: integrating genomic maps across species and data sources

**DOI:** 10.1186/1471-2105-14-246

**Published:** 2013-08-13

**Authors:** Trevor Paterson, Andy Law

**Affiliations:** 1 Division of Genetics and Genomics, The Roslin Institute, Royal (Dick) School of Veterinary Studies, University of Edinburgh, Easter Bush, Midlothian EH25 9RG, UK

**Keywords:** Software, Map drawing, Genetic map, Genomic map, Synteny, Conserved synteny, JEnsembl, Ensembl

## Abstract

**Background:**

The visualisation of genetic and genomic maps aligned within and between species and across data sources can be used to inform studies of genome evolution, assist genome assembly projects and aid gene discovery and identification. Whilst annotation, integration and exploration of assembled genome sequences is well supported, there are fewer tools available which can display genetic maps for less well-characterized species, and integrate these maps with annotated reference genomes to support cross species comparisons.

**Results:**

We have developed a desktop application to draw and align genetic and genomic maps, retrieved from remote data sources or loaded as local files. Maps can be retrieved from our public map database *ArkDB* or from any Ensembl data source (i.e. *Ensembl* and *Ensembl Genomes*). By using the JEnsembl API, maps can be drawn for any release version of any of the thousands of species present in Ensembl data sources, allowing not only inter-specific comparisons, but also comparisons between different versions/revisions of assembled genomes. Maps can be aligned by relating identical or synonymous markers across maps, or through the gene homology/orthology relationship data stored in the Ensembl Compara databases, allowing ready visualization of regions of conserved synteny between species. The map drawing canvas is highly configurable, supports interactive exploration of maps, markers and relationships and allows export of publication quality graphics.

**Conclusions:**

ArkMAP allows users to draw and interactively explore gene and variation maps for any version of any annotated genome curated in the Ensembl data sources, and to integrate local mapping data. The maps and inter-map relationships drawn are highly configurable and ArkMAP may be used to produce publication quality graphics. ArkMAP is freely available as an auto-updating Java ‘Web Start’ application, or as a standalone archived application.

## Background

The integration of genetic and physical maps, including annotated genome assemblies, is important for the investigation of genetic traits and the identification of candidate/target genes both in biomedical studies and in plant and animal breeding programmes. The interpretation of mapping data in one species can be assisted by integrating resources across species boundaries, where comparing genome structure and evolution can help identify potential gene homologues in regions of conserved synteny. This comparative genomic approach is particularly useful for the analysis of less well-characterized species by reference to the genomes of fully assembled and annotated genomes. However, there is a lack of flexible tool support for combining mapping data from disparate resources (i.e. local data, published mapping data and annotated genomes) especially where genetic maps cannot be represented in physical map coordinates. Furthermore, currently available tools have limited support for interactive exploration and relationship discovery between maps and species.

In recent years the bioinformatics community has developed a wide variety of tools for drawing maps and visualizing genomes initially to support genome assembly and browsing, and more recently to explore regions of conserved synteny between genomes (reviewed [1]). However, the alignment and comparison of larger chromosomal regions is a fundamentally different problem to that of aligning short sequences, with different visualization challenges. Particular problems include the difficulty of representing relationships between multiple chromosomes and multiple genomes at one time rather than as simple pairwise alignments, together with the problem of displaying different scales of data: from an ideographic overview of blocks of chromosome structure, down to the detailed ordering and orientation of the individual genes which have been aligned via sequence comparisons and calculated homology relationships [1].

The majority of ‘synteny browsers’ present a similar display model to that of the genome browsers, where pre-calculated sequence alignments or orthology relationships are used to highlight co-linear segments of different genomes relative to a reference genome. Most genome browsers use a horizontal layout of the genome assembly together with stacked horizontal annotation tracks which align genomic features (genes, transcripts, structural elements *etc*.) against the assembly sequence. Whilst genome browsers have been developed to support a single-species-centric data view, they may provide for the display of a limited amount of additional mapping data tracks, e.g. the location of genetic markers, and allow simple, *ad hoc*, user-defined maps to be added as tracks via DAS, GFF or bespoke data formats. In the case of the *UCSC* browser [2], pre-calculated blocks of inter-species conserved synteny can be displayed as annotation tracks against the reference genome; in *Ensembl*[3] pre-calculated conserved regions can be shown in tracks and pre-calculated, pair-wise blocks of synteny can also be displayed as a non-interactive overview map of (vertically) aligned chromosome regions for some pairs of species. The *NCBI MapView* browser [4] aligns maps vertically, but only provides orthology information between a limited number of the model species. Whilst ‘track-based’ data views allow the user to drill-down to the resolution of the nucleotide alignment, they do not provide a high level overview of the conservation of syntenic blocks at the level of the genome nor support interactive cross species comparisons. In addition, the server-side rendering of images and maps based on pre-calculated information held within these client/server data systems clearly limits the configuration options and interactivity.

Various alternative comparative map drawing tools are available, also built on a client server model, which typically requires non-trivial installation and configuration of server-side software and databases to allow users to draw configured maps in their web browser using their own or third-party-supplied data. For example, *SynBrowse*[5], *SynView*[6] and *GBrowse*_*syn*[7] are all based on the GBrowse [8] architecture, and as such limited to a particular GBrowse installation’s locally configured data sources for both the annotated genomes and inter-species orthology links. *Sybil*[9] is also part of the GMOD project, but stores genome data in a server-side Chado database allowing a web browser client to display synteny views and whole genome comparisons.

More generic map viewers often support the representation of alternative types of non-genomic mapping information, and may provide a wider choice of map-view models more appropriate to chromosome scale comparisons (for example vertical, ideographic or circular layout of maps as arcs). The *Sol Genomics Network* comparative map viewer [10] provides a limited choice of genetic and assembly maps from the Solanaceae family, but the user can also upload their own mapping data for comparison. The SGN server-side software is available and may be customized to display maps from other data sources. However, only a single pair of maps can be compared at one time. The popular *CMAP* software [11] can display and compare maps of any type and from any species and local installations are used by several genome projects. Again a CMAP server must be configured to use local databases of maps and tables of ‘correspondences’ (e.g. orthologies) between features on the maps.

Standalone map drawing software provides for yet more flexible map view display, and may also provide for the local computation of blocks of conserved synteny from input genome sequence and annotation files. The *SyMAP Synteny Browser*[12] is an example of such a system for identifying and displaying synteny alignments between chromosomal sequences. The synteny blocks from multiple chromosomes may be displayed in a high-level dot plot or three-dimensional view, from where the user can drill down to explore detailed alignments in a variety of views, including vertically drawn alignments. On the other hand *mGSV* (the *multi*-*Genome Synteny Viewer*) [13] uses local pre-calculated genome synteny data and annotation files to create a circular overview of inter-genomic relationships. Detailed multiple genome alignments are shown stacked horizontally, but with orthology relationships crossing over intervening genome tracks.

The circular layout of compared genomes (for example: *ChromoWheel*[14], *Circos*[15]) is claimed by many to provide a space-efficient and clear representation of inter-genomic relationships, because the relationship arcs do not transect the genome axes. On the other hand the circular layout seems to be more suited to displaying overview than local detail, and less readily allows for interactive repositioning and rescaling of individual maps. The *MizBee Multiscale Synteny Browser*[16] provides an elegant combination of interactive circular layout together with side-by-side views of comparative data across a range of scales.

We have developed ArkMAP as an alternative tool for aligning and drawing genetic and genomic maps. Because *ArkMAP* was developed as a tool for general biologists rather than for expert bioinformaticians it has been implemented as a standalone application which does not require installation of server-based software or databases. In order to avoid the requirement for local data sources ArkMAP accesses mapping data by default from the publicly available Ensembl systems (Ensembl Vertebrates [3] and Ensembl Genomes [17]). The Ensembl data sources hold thousands of annotated genome assemblies including those from vertebrates, invertebrates, metazoa, protists, fungi, plants and bacteria. In addition to the core assembly and gene annotation data for each species, Ensembl may hold a variety of further information resources including variation data and gene homology relationships to other species which allows for the integration of data across species boundaries. ArkMAP also provides for the integration of personal mapping data of any type (i.e. sequence, linkage, radiation hybrid, cytogenetic *etc*.) by uploading plain text or XML formatted files, and provides access to the ArkDB database of genetic and cytogenetic maps [18]. The ArkDB system was developed as a curated repository of mapping data for 10 farm animal species, but has been extended to allow registered users to archive (and retrieve) arbitrary personal mapping data of any type for any species.

Although ArkMAP uses mapping data accessed remotely because the maps are rendered locally the drawing canvas is highly configurable and interactive, allowing drag and drop repositioning of maps as well as the ability to filter maps, markers and relationships as required. In order to provide the most efficient and configurable layout of the maps, marker labels and inter-map relationship arcs, ArkMAP uses a vertical alignment of maps on the canvas. In addition to displaying homology relationships retrieved from Ensembl, mapped features can be related across maps using either synonymy (shared marker names) or identity (shared accession identifiers) thus providing true integration of local mapping data with the public genome assembly resources.

## Implementation

ArkMAP was originally implemented as a desk-top replacement for the applet-based drawing of maps used by the ArkDB web-application, launching automatically through Java Web Start technology and presenting data solely from that data source. It has now been developed as a more versatile standalone application that can: import mapping data from a variety of sources, align maps on the basis of inter-marker relationships, allow retrieval of extra information about maps and markers (e.g. variation data, homology information, assembly and annotation versions) and provide a drawing canvas with an interactive and space-efficient layout that allows reconfiguration, rescaling and repositioning of maps and inter-map relationships. In addition ArkMAP can export publication quality images of aligned maps. ArkMAP is also available as a simple downloadable application as an alternative for users who experience problems with Web Start compatibility, although the bundled version does not provide auto–updating.

ArkMAP’s functionality can be divided into three aspects: data retrieval and integration, map drawing and layout, and relationship discovery and representation. No attempt is made to impose rules on what constitutes a “block of conserved synteny” and the data and relationships displayed derive from analyses conducted elsewhere e.g. based on assertions of orthology held within the Ensembl system.

ArkMAP is built re-using code libraries developed for the ArkDB web application and web services, representing genetic objects (species, chromosomes, markers, maps *etc*.) in a ‘model layer’. The application user interface and map drawing code is implemented using the standard Java Swing GUI libraries, which represent map objects through a ‘view layer’. Integration of the JEnsembl API libraries [19] provides access to data in Ensembl data sources, with ArkMAP converting objects from the JEnsembl genetic model to the ArkDB model.

### Data retrieval

ArkMAP menu options allow the user to browse, download and draw available maps from ArkDB, Ensembl Vertebrates, Ensembl Other Genomes and Ensembl Bacteria, to open local GMD-compliant XML files of mapping data (GMD: GenomicMappingData.xsd [20]) or to create new maps using plain-text entry (which also allows for local text-file upload). The ArkDB web application provides SOAP web services which expose mapping data according to a standard (GMD) XML schema. Serialized map data is bound by Java and represented according to the ArkDB data model using the ArkDB binary libraries. Local XML files can be parsed using the same libraries. All connectivity to Ensembl MySQL databases is provided by the JEnsembl API. The JEnsembl configuration module ensures that evolving versions of the Ensembl database schema are handled correctly and provides backwards compatibility to previous release versions of the schema. Updates to this configuration are required for each new Ensembl release in order to incorporate future schema alterations. The synchronisation of JEnsembl’s configuration is provided by Web Start’s auto-updating feature, which will automatically download the most recent ArkMAP version.

Because – in their most basic form – maps are essentially simple lists of mappable objects (RFLPs, repeat regions, PCR probes, genes, sequences etc.) located on arbitrary co-ordinate systems, any data set can be expressed in usable form for ArkMAP either via textfile upload or by deposition of the data into the ArkDB system. For example, sequences of primer pairs used to generate marker genotype data within a genetic linkage map can readily be mapped against specific sequence assemblies using ePCR and the resulting lists of hit locations used as a “bridge” between genetic linkage and sequence assembly maps. Several ePCR maps are already stored within the ArkDB system where they were constructed by using the EMBOSS ‘primersearch’ program [21] to map ArkDB primer pairs from various curated analyses to the genome assemblies provided by Ensembl. Multiple hits and hits to non-assembled fragments and contigs were discarded, and the remaining unique ePCR mapping positions curated in ArkDB as ePCR sequence maps for each chromosome (for Pig, Cow, Chicken and Turkey). Maps retrieved from Ensembl use the chromosomal base pair coordinate system defined in that database, whereas genetic linkage maps from ArkDB utilise the map location specified in cM from the published data. Because each map is self-contained, each is free to implement its own numerical coordinates. Thus even simple “chromosome-painting” syntenic block data can be displayed and subsequently “joined” to sequence assembly data through synonomy relationships.

### Map display

The ArkMAP GUI window contains an extendible drawing canvas where downloaded maps are displayed. The maps are drawn in vertical orientation which allows a more efficient and flexible use of the drawing panel area, particularly because marker labels are written horizontally. Each map can be freely repositioned on the canvas by mouse dragging, and may be resized and zoomed. Collapsible GUI configuration panels provide widgets for maps to be hidden, deleted or renamed and the user can sensitively control the level of detail displayed for each map and the overall canvas layout by individually configuring marker labels and a variety of display options.

Various data retrieval and configuration wizards are available from the ArkMAP main menu, or from a ‘Context Menu’ particular to an individual map, and presented by selecting a region of a map’s axis. This ‘Context Menu’ allows a user to zoom or project (duplicate) the selected map region. The ‘Context Menu’ also provides the starting point to retrieve related maps for a selected region of the map. For ArkDB-derived ePCR maps, the ‘Context Menu’ allows the user to import the cognate Ensembl chromosome assembly for the selected map region, whilst for Ensembl assembly maps the user can import alternate release versions of the annotated assembly, dbSNP variation maps (where available) and search for regions of conserved synteny in specified target species.

### Marker information

Hovering over a marker name displays its actual mapping coordinates, whilst selection by means of mouse-clicking displays a ‘Marker Detail’ window reporting additional marker information such as the database accession or stable ID, the marker type and its description. Further navigation options presented in this window depend upon the data source: ArkDB markers can be searched for all other mappings of the marker on all maps in the database, whilst for Ensembl derived markers (genes and SNP variants) the user can choose to open an Ensembl browser window at the relevant genomic location or perform a homology search for the selected gene. This latter functionality is implemented by the JEnsembl API, which searches the appropriate Ensembl Compara data source for curated gene homologies. The results are presented in a detailed table format.

Where available, for example for the human genome assembly, a third type of marker may be displayed on Ensembl maps: ‘Assembly Exceptions’. These alternate haplotypes and assembly patches between genome builds are drawn as a special type of colour-highlighted marker. In this case the ‘Marker Detail’ window provides for import from Ensembl of an annotated map of the whole exception region.

### Displaying relationships

By default ArkMAP is configured to draw relationship links between markers with either shared database identifier (*identity*) or shared name (*synonymy*). Relationships are only drawn between maps placed in adjacent, *virtual* columns on the drawing canvas. Columns are automatically calculated from the map positions on the canvas, and may contain more than one map, where the maps’ width boundaries overlap. By careful repositioning of maps on the canvas the user can finely control between which maps relationships are drawn.

Currently the only other types of inter-marker links drawn by ArkMAP are colour-coded homology relationships between Ensembl genes. These relationships are created when regions of conserved synteny are downloaded for a selected map region using the ‘Context Menu’ as described above. Homology relationships are classified by the Ensembl Homology pipeline using a gene orthology/paralogy prediction method [22]. The default colour palette is chosen for maximum resolution by colour blind users (following CUDO guidelines [23]), but the user may select their own colour scheme. The JEnsembl API also retrieves amino acid similarity and identity scores for homologous gene pairs and these values can be used to filter the sensitivity of link reporting. Minimum display thresholds can be set with an interactive slider bar, for either the identity or similarity scores of homology relationships. The user may choose to set reporting stringency on individual maps (again through the ‘Context Menu’) or globally across the whole application.

The architecture and data model of the ArkMAP application allows for future integration of additional data resources. For example it would be possible to provide ‘drill-down’ features to retrieve additional information about markers, and it may be possible to create further types of marker relationships, and to filter on different properties of relationships.

### Running ArkMAP

As described above, the auto-updating Web Start installation is available at the ArkMAP homepage [24] together with full instructions for running and configuring ArkMAP. Because drawing large genome assemblies with potentially thousands of genes and relationship is memory intensive, Java should be configured to utilize as high memory as possible. Potential installation problems with Java Web Start on Windows machines are addressed in the help file. We now also provide a Launch4J installer bundle as an alternative to Web Start, which will install the appropriate 32- or 64-bit version of the ArkMAP program. This bundled version does not provide for auto–updating, but will display a warning dialog when out of date with the ArkMAP version currently available.

At any point the user can export a screen shot of the drawing canvas as an SVG graphics file or a PDF. The canvas can also be printed, using on-screen guides to help organize pagination, and any maps that the user has created locally can be exported in GMD XML format.

Detailed online documentation is available through a browser link in the ‘Help’ menu and covers issues concerning ArkMAP installation, downloading maps and configuring the display of map and inter-map relationships.

## Results and discussion

The Web Start version of ArkMAP is now the default tool used to display maps in the ArkDB web application, replacing applet drawing code. This not only allows more flexible configuration options for the user, but also allows connection to third party data resources which was not possible from within a sandboxed applet.

When accessed from ArkDB, ArkMAP is automatically preloaded with the user’s selected maps, otherwise maps are downloaded via the drop down ‘File’ menu: local XML files may be opened, new maps created with a text editor, or the user may download maps from one of the configured data sources (ArkDB, Ensembl Vertebrates, Ensembl Genomes and Ensembl Bacteria). By selecting a data source the user can browse and select maps for available species, analyses or Ensembl releases. The same File menu allows screenshots of the canvas to be saved at any time as SVG, PDF or PNG graphics files, or printed locally.

**Figure 1:**
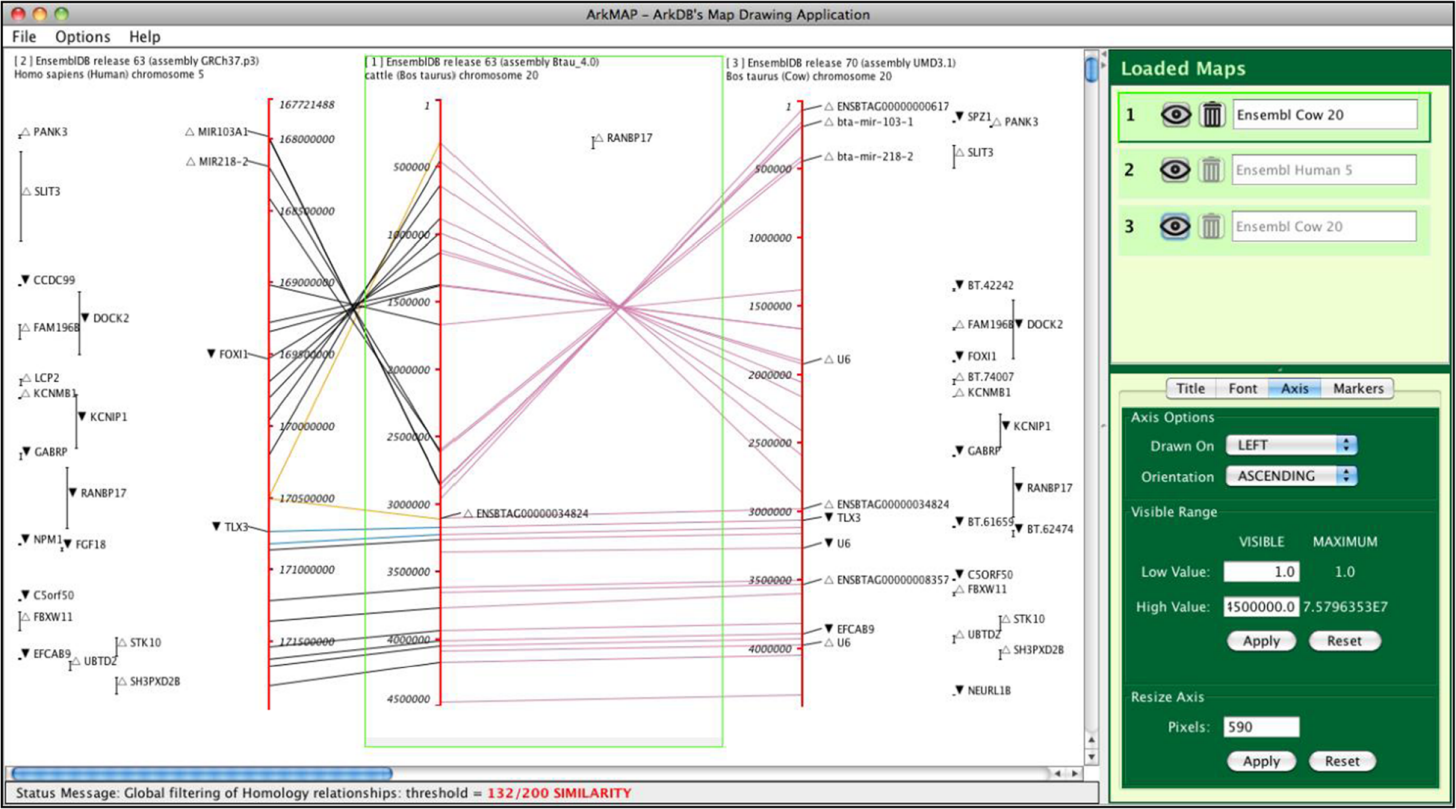
**Screen shot of ArkMAP showing three gene maps uploaded from Ensembl.** Map 1 (highlighted) of Cow chromosome 20 was retrieved from Ensembl release 63. Map 2 was retrieved by searching for human regions of conserved synteny to map 1. Map 3 was retrieved by fetching the more recent release version 70 for the region displayed in map 1. The upper configuration panel allows the selected map to be hidden or deleted, and the tabbed panels below allow rescaling, resizing, reorienting of the axis; changing the map title; choosing font types and sizes and selecting which markers to display. Colour key: purple: *identity*, sky-blue: *synonymy*, black: *one*-*to*-*one orthology*, vermilion: *apparent one*-*to*-*one orthology*, orange: one-to-many orthology, blue-green: *many*-*to*-*many orthology*, blue: *possible orthology*, yellow: *paralogy*. ▲ forward transcribed gene; ▽reverse transcribed gene.

**Figure 2:**
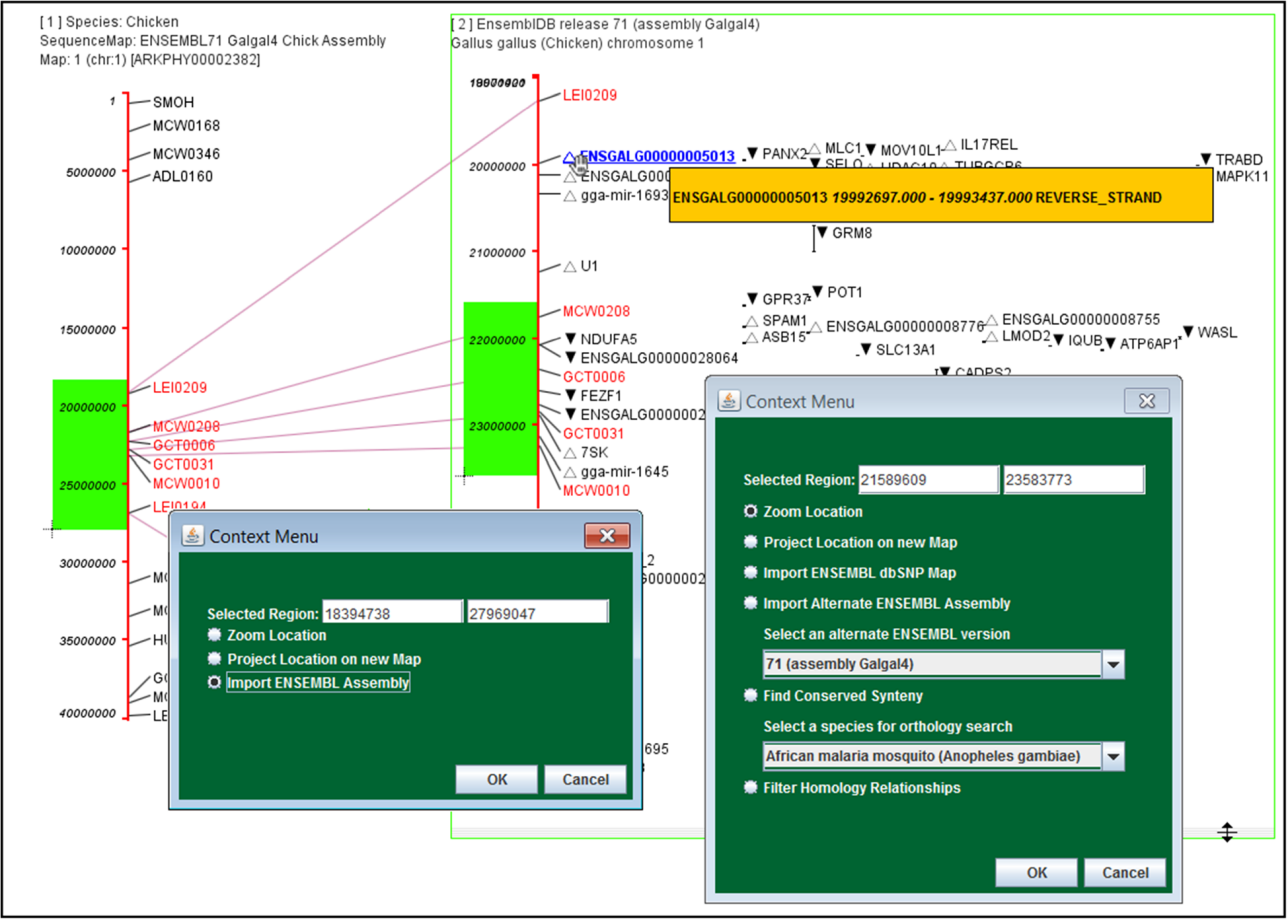
**Composite screen shot of ArkMAP illustrating various interactive options.** Map 1 has been zoomed to show bases 1-40M of a chicken ePCR map from ArkDB, the selected (green highlighted) region of this map has been used to retrieve the cognate gene-annotated assembly from ENSEMBL (map 2). As the mouse cursor is moved over the canvas it changes to reflect available actions for the underlying map object. Three versions of the cursor are shown here: cross hairs used to select regions on the axes, a double arrow used to stretch the selected map (2) and the open hand indicating that the map can be moved by mouse dragging. In addition the (hand) cursor has triggered display of a pop-up box displaying the coordinates of the blue-highlighted marker. Mouse clicking this selected marker would display a more detailed pop-up with further information and interaction options. The gene mappings from Ensembl include orientation information: ▲ forward or ▽ reverse transcribed gene. Alternate ‘Context Menus’ are shown for selected regions of each map. In both cases the user may zoom or project (duplicate) the selected map region. In the case of the ArkDB ePCR map the user may choose to import the cognate Ensembl chromosome (performed here), whilst for the Ensembl assembly map the user may import alternate release versions of the annotated assembly, import dbSNP variation maps (where available), search for regions of conserved synteny in a specified target species or choose to perform threshold filtering on any homology relationships for genes on this map.

**Figure 3:**
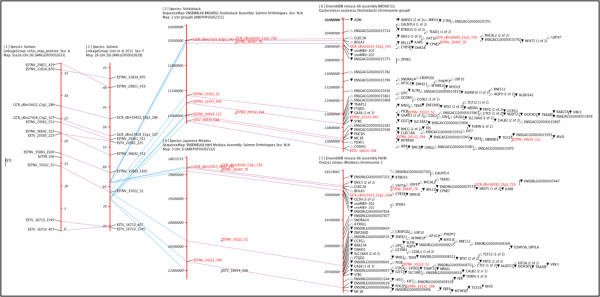
**Composite screen shot of ArkMAP detailing a mapping analysis of low quality Salmon data.** Map 1 is a private (and obfuscated) salmon QTL linkage map (Houston RD, Gonen S: *per*.*comm*.) which uses SNP markers shared on published linkage map of salmon chromosome 26 (map 2 [25]). The best BlastX hits for sequences flanking the salmon SNPS were mapped as ‘orthologous sequences’ on the stickleback (map 3) and medaka (map 4) genome assemblies. These maps were then projected onto the gene annotated assemblies downloaded from Ensembl (maps 5 and 6). The figure is a composite screenshot from ArkMAP showing ‘Identity’ relationships in purple and ‘Synonymy’ relationships in blue (key as in Figure 1).

The ArkMAP user interface is dominated by the drawing canvas upon which maps can be freely positioned and resized. The canvas is scrollable and automatically expands as new maps are added. Maps are sensitive to mouse actions, and on mouse entry the rectangular bounds of a map are drawn in grey; selecting a map by clicking within this area changes the border to green; grey stretch bars at the bottom allow the map to be resized by mouse dragging (see Figures 1 and 2). Maps may be dragged around the canvas and because maps are transparent they may overlap each other, allowing the user fine control of image composition. Maps may be stacked or even overlapped in ‘columns’, and because inter-marker relationships are only drawn between maps in adjacent ‘columns’ the user can sensitively control what information is visible (in Figure 3 maps 3 & 4, and maps 5 & 6 are in adjacent columns).

When a map is selected various (collapsible) configuration panels are displayed (see Figure 1). In the upper panel maps may be hidden (using the ‘eye’ icon) or permanently removed. Separate tabbed control panes allow rescaling, resizing, reorienting of the axis; changing the map title; choosing font types and sizes and selecting which markers to display.

Maps are drawn vertically as a scaled red axis, with labelled markers positioned as points or ranges; where available marker orientation is shown (see Figures 1 and 2). The user can select a sub-region of a map by mouse dragging along the labelled side of the axis, causing a context-dependent menu to be displayed beside the highlighted selection (see Figure 2). As described above, in addition to facilitating zooming or duplication (projection) of the region, various further options are presented according to type of map.

Figure 1 illustrates an ArkMAP workflow that compared bovine genome builds between Ensembl releases, which was informed by querying a map for regions of conserved synteny in a better characterized model species (human). The gene-annotated map of bovine chromosome 20 (map 1) was loaded from Ensembl release 63 which used assembly version Btau_4.1. This map has been zoomed to show the first 4.5 MB, and the majority of marker labels have been hidden using the configuration panel. This region was selected, and searched via the ‘Context Menu’ for regions of conserved synteny with the annotated human genome. Conservation queries are performed by the JEnsembl API, which successively searches the Ensembl Compara data source for human homologues to each bovine gene located on the selected region. It should be noted that as the search time is proportional to the number of genes selected, searching large chromosomal regions may cause ArkMAP to become non-responsive. The orthologous region of human chromosome 5 (map 2) has been aligned with the bovine map, with the gene homology relationships represented by lines coloured according to type of homology; in this case the user configured the human map to label only those genes homologous to cattle genes. The alignment clearly shows an apparent inversion in the distal portion of cow chromosome 20 relative to the human gene order, with Ensembl classifying the majority of homology relationships as 1:1 orthologies. Note that the ‘Status Message’ panel records the current threshold used for filtering homologies (controlled from the ‘Options’ menu).

The same region of map 1 was used to identify and import the corresponding region from a more recent annotated version of the bovine genome from Ensembl release 70, which used the UMD3.1 genome assembly. This map (3) has been aligned alongside the Btau_4.0 assembly with the genes sharing stable Ensembl identifiers linked in purple (again the UMD3.1 map has been configured to only label genes related to those present on the Btau_4.0 assembly). We can clearly see that the inversion has been ‘corrected’ in the more recent assembly build, to match the human gene order. Interestingly the human gene RANBP17 is shown to be (1:N) orthology linked to two cattle genes bounding the potential ‘inversion’ or assembly error. The full gene description which is reported (not shown) when clicking the ‘novel’ bovine gene ENSBTA000000034824 categorizes this marker as a processed pseudogene, which may indicate a possible source of the ambiguity.

Figure 2 illustrates some of the interactive features available in ArkMAP, as described in its legend. Of particular note is how the selection of a region of a map leads to the presentation of a context-dependent menu of possible actions that may be performed using the chosen map coordinates and the encompassed markers.

Figure 3 illustrates how ArkMAP may be used to integrate personal mapping data, with data curated by the ArkDB and Ensembl resources. In this case a local map (1) has been created positioning a salmon QTL marker on a linkage map of chromosome 26. This map is aligned (by marker identity) with a published salmon linkage map [25] (map 2), downloaded from a private ArkDB data source. The salmon SNP markers were analysed by BlastX in order to map orthologous sequence markers on the genome assemblies of stickleback and Japanese medaka. Chromosome regions sharing markers (by synonymy) with the QTL region of interest were downloaded, zoomed and aligned with the salmon linkage map (maps 3 and 4). Using the ‘Context Menu’ these regions of the physical maps were used to retrieve the cognate regions of the stickleback and medaka genome assemblies from Ensembl (transferring the ‘salmon orthologue’ markers across to the gene-annotated maps; shown in red, maps 5 and 6). The gene maps for the better characterized model fish species are therefore now aligned with a potential region of conserved synteny in the poorly characterized salmon QTL region. The user could extend the analysis by aligning synonymous markers on the medaka and stickleback maps, querying for regions of conserved synteny between various fish genomes, and drilling down to get details and homologies for individual genes on the maps.

It is worth noting here that care should be taken when considering the significance of synonymy relationships. Sharing a name does not necessarily imply that the markers are the same, use an identical detection technique or are orthologous between species. Conversely if markers on different maps have the same ArkDB accession ID or Ensembl stable identifier, there is greater confidence that they represent the same object on each map (e.g. for ArkDB a marker typically represents a particular detection protocol, whereas Ensembl IDs represent a stable curated gene model). In the analysis shown in Figure 3, the markers present on the ArkDB stickleback and medaka maps are Blast analysis target hits, which have been named identically to the source salmon sequences, so we can be reasonably confident in the significance of the ‘synonymy’ relationships between the salmon, stickleback and medaka maps.

**Figure 4:**
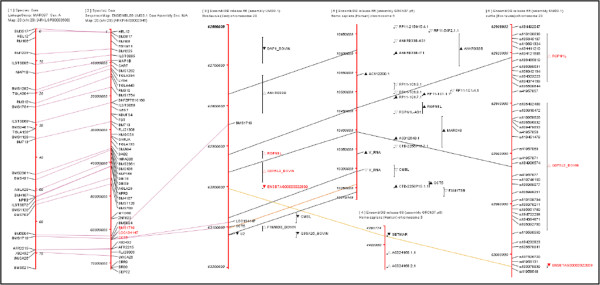
**Drawing canvas exported from ArkMAP detailing a cow mapping analysis.** Map 1 is a legacy linkage map downloaded from ArkDB. Many of the microsatellite markers are located on ePCR map also in ArkDB (map 2). A region of interest has been projected onto the gene annotated chromosome map from Ensembl (map 3) and this region searched for human regions of conserved synteny (maps 4 and 5). Map 6 shows some of the SNP variation markers retrieved from Ensembl for the bovine region of interest (key as in Figure 1).

Figure 4 illustrates the integration of public mapping data across the ArkDB and Ensembl sources. In this case a legacy bovine linkage map (1) is aligned by marker identity with an ePCR map of the marker set on the bovine genome (2). Both of these maps are downloaded from ArkDB, and because map2 uses an Ensembl genome assembly, a region of interest can be used to download the cognate gene-annotated chromosome map from the same Ensembl release version (3), again projecting the ArkDB markers onto the Ensembl map. The region interest selected on map 3 (9 cattle genes) is used to search for regions of conserved synteny in the human assembly. A collinear region of conservation on human chromosome 5 (map 5) carries orthologues of 6 of these genes, but has a far larger number of annotated genes (probably reflecting the far fuller state of analysis for the human genome). A sub-region of map 3 has been selected to download SNP-variant mappings from the Ensembl Variation data source available for cattle, drawn as map 6.

The preceding examples illustrate how ArkMAP can be used as a flexible tool to draw and relate arbitrary maps of chromosomes or chromosome regions for any species annotated in the publicly available Ensembl data sources. It is trivial to upload local mapping data, and hence integrate private genetic maps with the publicly available data.

Future developments of the software might include provision of the facility to upload mapping data in other formats (GFF *etc*.) or the ability to configure connection to local private Ensembl instances. It might also be productive to add the ability to mine more information from the Ensembl databases, perhaps including phenotype data and external references to third party data sources. Adding the capability to connect to additional styles of data sources (i.e. non-Ensembl, non-GMD compliant) would require additional parsing/conversion modules to be added to the ArkMAP architecture, but could have great value for certain user groups.

## Conclusions

We have built a freely available desktop tool for drawing and integrating genomic and genetic maps for any species, capable of integrating data from remote Ensembl, ArkDB and local data sources. Uniquely this allows traversal from data poor maps in one species to well annotated regions of conserved synteny in model organisms, via the gene orthology relationships curated in Ensembl. This allows the characterisation of chromosome regions of interest and the discovery of potential new target genes associated with genetic traits. ArkMAP can be used to visualize genome assembly evolution, and can be used to help resolve assembly ambiguities by reference to better characterized species. ArkMAP can also be used to visualise chromosomal evolution as blocks of conserved synteny. ArkMAP provides a unique visualisation of the interspecies homology information held in Ensembl Compara data sources, and the flexible layout for drawing maps allows for interactive exploration of inter-map relationships and the production of publication quality graphics.

## Availability and requirements

**Project name**: ArkMAP

**Project Home Page**: http://bioinformatics.roslin.ed.ac.uk/arkmap/

**Operating System**: platform independent

**Other Requirements**: Java Runtime Environment version 1.6+

**Usage Restrictions**: none

## Competing interests

The authors declare that they have no competing interests.

## Authors’ contributions

AL conceived the project and oversaw its design. TP developed ArkMAP and implemented the ArkDB web services building on existing ArkDB architecture developed by the ArkDB project team (see Acknowledgements below). TP drafted the manuscript and both authors read and approved the final manuscript.
